# GABAergic neurons in the preoptic area send direct inhibitory projections to orexin neurons

**DOI:** 10.3389/fncir.2013.00192

**Published:** 2013-12-02

**Authors:** Yuki C. Saito, Natsuko Tsujino, Emi Hasegawa, Kaori Akashi, Manabu Abe, Michihiro Mieda, Kenji Sakimura, Takeshi Sakurai

**Affiliations:** ^1^Department of Molecular Neuroscience and Integrative Physiology, Faculty of Medicine, Kanazawa UniversityKanazawa, Japan; ^2^Department of Cellular Neurobiology, Brain Research Institute, Niigata UniversityNiigata, Japan

**Keywords:** orexin, preoptic area, GABA, sleep, wakefulness, hypothalamus

## Abstract

Populations of neurons in the hypothalamic preoptic area (POA) fire rapidly during sleep, exhibiting sleep/waking state-dependent firing patterns that are the reciprocal of those observed in the arousal system. The majority of these preoptic “sleep-active” neurons contain the inhibitory neurotransmitter GABA. On the other hand, a population of neurons in the lateral hypothalamic area (LHA) contains orexins, which play an important role in the maintenance of wakefulness, and exhibit an excitatory influence on arousal-related neurons. It is important to know the anatomical and functional interactions between the POA sleep-active neurons and orexin neurons, both of which play important, but opposite roles in regulation of sleep/wakefulness states. In this study, we confirmed that specific pharmacogenetic stimulation of GABAergic neurons in the POA leads to an increase in the amount of non-rapid eye movement (NREM) sleep. We next examined direct connectivity between POA GABAergic neurons and orexin neurons using channelrhodopsin 2 (ChR2) as an anterograde tracer as well as an optogenetic tool. We expressed ChR2-eYFP selectively in GABAergic neurons in the POA by AAV-mediated gene transfer, and examined the projection sites of ChR2-eYFP-expressing axons, and the effect of optogenetic stimulation of ChR2-eYFP on the activity of orexin neurons. We found that these neurons send widespread projections to wakefulness-related areas in the hypothalamus and brain stem, including the LHA where these fibers make close appositions to orexin neurons. Optogenetic stimulation of these fibers resulted in rapid inhibition of orexin neurons. These observations suggest direct connectivity between POA GABAergic neurons and orexin neurons.

## Introduction

The preoptic area (POA) of the hypothalamus has been implicated in a variety of physiological functions, including the regulation of sleep/wakefulness states (Boulant, 1981; McGinty et al., 2001). Especially, this region is thought to play an important role in the initiation and maintenance of sleep. Initially, electrical or chemical stimulation of the lateral POA in animals was shown to promote EEG slow-wave activity and sleep onset (Sterman and Clemente, 1962; Benedek et al., 1982; Ticho and Radulovacki, 1991; Mendelson and Martin, 1992). Consistently, lesions in the POA have been shown to result in profound and persistent sleep loss (John and Kumar, 1998; Lu et al., 2000). However, these studies are not genetically targeted to specific neuron types, and so the cells that are responsible remain to be clarified. In this study, we focused on the GABAergic neurons, as some of them have been shown to provide inputs in particular to the arousal system. We examined the role of the projection from GABAergic neurons in the POA to the orexin neurons in the LHA in inhibiting the latter cells, and the effect of activating this pathway on sleep.

Extracellular recording studies have identified sleep-active neurons in a region extending from the medial through the lateral POA (Kaitin, 1984; Koyama and Hayaishi, 1994), while it was reported that neurons in the rat ventrolateral preoptic area (VLPO) and median preoptic nucleus (MnPN) exhibited Fos expression following consolidated sleep (Gong et al., 2000). The POA was shown to send GABAergic inhibitory projections to monoaminergic regions, including the locus coeruleus (LC), dorsal raphe nucleus (DRN), and tuberomammilary nucleus (TMN) (Sherin et al., 1996, 1998; Steininger et al., 2001; Uschakov et al., 2007). Consistently, firing patterns of monoaminergic neurons in these nuclei across the sleep-waking cycle are the reciprocal of those observed in POA sleep-active neurons. They fire at a rapid rate during wakefulness, slow down during non-rapid eye movement (NREM) sleep, and cease firing during rapid eye movement (REM) sleep (Saper et al., 2001). Electrophysiological studies suggested that VLPO neurons are inhibited by noradrenaline and serotonin (Gallopin et al., 2000), suggesting mutually inhibitory interactions between VLPO and the monoaminergic arousal systems (Saper et al., 2001).

Monoaminergic arousal systems are also thought to be regulated by orexin neuropeptides, which are thought to be a critical regulator of sleep/wake states (Sakurai, 2007). Orexin deficiency causes the sleep disorder narcolepsy in humans and animals (Chemelli et al., 1999; Lin et al., 1999; Peyron et al., 2000; Thannickal et al., 2000; Hara et al., 2001). Orexin-producing neurons (orexin neurons) in the lateral hypothalamic area (LHA) send dense axonal projections to monoaminergic neurons in the brain stem/hypothalamic regions. Recent studies have suggested the POA also sends projections to orexin neurons in the LHA (Sakurai et al., 2005; Yoshida et al., 2006). However, how the endogenous firing of POA sleep-active neurons affects the activity of orexin neurons has been unknown. Functional studies of this issue are important, because both POA sleep active neurons and orexin neurons play highly important roles in the physiological regulation of sleep.

To examine the electrophysiological impact of activity of endogenous POA neurons, we first confirmed that selective pharmacogenetic stimulation of GABAergic neurons in the POA, using the Designer Receptors Exclusively Activated by Designer Drugs (DREADD) technology, leads to an increase of NREM sleep (Armbruster et al., 2007). We also used channelrhodopsin-2 (ChR2) as an anterograde tracer as well as an optogenetic tool (Bernstein et al., 2012; Yizhar et al., 2011) for selective optical excitation of GABAergic POA neurons and their axons. We examined the axonal projections of ChR2-eYFP-positive fibers and confirmed that POA GABAergic neurons send abundant projections to arousal-related regions, including the LC, DR, TMN, and laterodorsal/pedunculopontine tegmental nucleui (LDT/PPT). Dense projections were also found in the LHA, and these GABAergic fibers made appositions to orexin neurons. We then explored the effects of fast and selective optogenetic stimulation of GABAergic axons on orexin neurons. By combining whole-cell patch-clamp recordings from orexin neurons with optogenetic stimulation of GABAergic axons in acute mouse brain slices, we found that photostimulation of POA GABAergic fibers immediately caused a decrease in the firing rate of orexin neurons through GABA release. These observations suggest that POA GABAergic neurons send direct inhibitory projections to orexin neurons.

## Materials and methods

### Animals

All experimental procedures involving animals were approved by the Animal Experiment and Use Committee of Kanazawa University (AP-132649), and were thus in accordance with NIH guidelines. *Gad67-Cre* mice, in which the *Cre* gene was knocked-in in the *Gad67* allele were previously described (Wu et al., 2011). The mice were bred with wild type C57BL/6J mice more than ten times and maintained.

### AAV production and purification

We used AAV with the FLEX switch system (Atasoy et al., 2008) to specifically express HA-tagged hM3Dq or ChR2 fused with EYFP (ChR2-EYFP) only in Cre recombinase-expressing neurons. We applied this method to heterozygous *Gad67-Cre* mice in which the Cre recombinase gene is specifically expressed in GABAergic neurons (Wu et al., 2011).

*pAAV-DIO-HAhM3Dq* was provided by Dr. Brian Roth. *pAAV-DIO-hChR2(H134R)-EYFP-WPRE-pA* was provided by Dr. Karl Deisseroth of Stanford University (Kozorovitskiy et al., 2012). We constructed a plasmid, *pAAV-horexin-tdTomato-WPRE-pA*, as follows. A 1.3-kb fragment of the human *prepro-orexin* gene promoter, which has the ability to drive expression in orexin neurons specifically (Moriguchi et al., 2002), was amplified by PCR with a pair of primers 5′-CACGCGTGCATGCTGTAATCCCAGCTAC-3′ and 5′-TGTCGACGGTGTCTGGCGCTCAGGGTG-3′. The PCR product was fully sequenced and digested by MluI and SalI, and ligated to Mlu I and SalI-digested *pAAV-DIO-hChR2(H134R)-EYFP-WPRE-pA*, yielding *pAAV-horexin-hChR2(H134R)-EYFP-WPRE-pA*. The *tdTomato* gene fragment from *ptdTomato* (Clontech) was inserted into the EcoRI and SalI sites of *pAAV-horexin-hChR2(H134R)-EYFP-WPRE-pA*, yielding *pAAV-horexin-tdTomato-WPRE-pA*.

Viruses were produced using a triple-transfection, helper-free method using a modification of a published protocol (Auricchio et al., 2001; Sasaki et al., 2011). The final purified viruses were aliquoted and stored at −80°C. The titers of *AAV-DIO-hChR2(H134R)-EYFP* and *AAV-orexin-tdTomato* were 1.63 × 10^12^ and 1.03 × 10^12^ genome copies/ml, respectively.

### Virus injection

Adenoassociated-virus *AAV-DIO-HAhM3Dq* or *AAV-DIO-hChR2(H134R)-EYFP* was injected into the POA of *Gad67-Cre* mice (Wu et al., 2011). In *in vitro* electrophysiological experiments for recording orexin neurons, *AAV-orexin-tdTomato* was simultaneously injected into the LHA of these mice for identification of orexin neurons (Figure 4A). Male mice were anesthetized with isofluorane and placed in a stereotaxic frame (David Kopf Instruments). For injection into the POA, two holes were drilled into the skull of *Gad67-Cre* mice (12–15 weeks of age, weight 25–30 g), at sites +0.3 mm anterior, ±0.65 mm lateral, and −5.72 mm ventral to the bregma under deep anesthesia. For injection into the LHA, four holes were drilled into the skull of each mouse under anesthesia, at sites −1.4 mm posterior, ±0.9 mm lateral, and −5.5 mm ventral; and −1.8 mm posterior, ±0.9 mm lateral, and −5.7 mm ventral to the bregma (four injection sites per mouse).

A Hamilton needle syringe (33-gauge) was placed at each site, and 0.5 μl purified virus was delivered to each site over a 10-min period. After 5 min of rest, the needles were removed. The mice were sacrificed 14 days later, and slice preparations were analyzed by electrophysiological experiments and tissue samples by immunohistochemical staining.

### Electrophysiology

Acute slices containing the LHA were prepared from the mice 14 days post-AAV injection, as described in our previous studies (Tsujino et al., 2005). The mice were decapitated under deep anesthesia. Brains were isolated in ice-cold cutting solution consisting of (mM): 280 sucrose, 2 KCl, 10 HEPES, 0.5 CaCl_2_, 10 MgCl_2_, 10 glucose, pH 7.4, bubbled with 100% O_2_. Brains were cut coronally into 300-μm slices with a vibratome (VTA-1200S, Leica, Germany). Slices were transferred to an incubation chamber at room temperature filled with physiological solution containing (mM): 125 NaCl, 2.5 KCl, 1.25 NaH_2_PO_4_, 2.0 CaCl_2_, 1.0 MgSO_4_, 26 NaHCO_3_, 11 glucose, pH 7.4, bubbled with 95% O_2_/5% CO_2_. After 1-h incubation in an incubation chamber, the slices were transferred to a recording chamber (RC-27L, Warner Instrument Corp., CT, USA) at 32°C on a fluorescence microscope stage (BX51WI, Olympus, Tokyo, Japan). Neurons that showed tdTomato fluorescence were used for patch-clamp recordings. The fluorescence microscope was equipped with an infrared camera (C-3077, Hamamatsu Photonics, Hamamatsu, Japan) for infrared differential interference contrast (IR-DIC) imaging and a CCD camera (JK-TU53H, Olympus) for fluorescent imaging. Each image was displayed separately on a monitor. Recordings were carried out with an Axopatch 200B amplifier (Axon Instruments, Foster City, CA) using a borosilicate pipette (GC150-10, Harvard Apparatus, Holliston, MA) prepared using a micropipette puller (P-97, Sutter Instruments, Pangbourne, UK) and filled with intracellular solution (4–10 MΩ), consisting of (mM): 125 K-gluconate, 5 KCl, 1 MgCl_2_, 10 HEPES, 1.1 EGTA-Na_3_, 5 MgATP, 0.5 Na_2_GTP, pH7.3 with KOH. Osmolarity of the solution was checked with a vapor pressure osmometer (model 5520, Wescor, Logan, UT). The osmolarity of the internal and external solutions was 280–290 and 320–330 mOsm/l, respectively. The liquid junction potential of the patch pipette and perfused extracellular solution was estimated to be −16.2 mV and was applied to the data. The recording pipette was under positive pressure while it was advanced toward individual cells in the slice. Tight seals of 0.5–1.0 GΩ were made by applying negative pressure. The membrane patch was then ruptured by suction. The series resistance during recording was 10–25 MΩ and was compensated. The reference electrode was an Ag-AgCl pellet immersed in bath solution. During recordings, cells were superfused with extracellular solution at a rate of 1.0–2.0 ml/min using a peristaltic pump (K.T. Lab, Japan).

Light activation was performed using an LED device (KSL-70; Rapp OptoElectronic, Hamburg, Germany) at a wavelength of 470 nm (maximum: 8 mW/mm^2^). Pulse was generated with SEN-3301 stimulator (Nihon Koden, Japan).

### Clozapine-N-oxide administration

Clozapine N-oxide (CNO; C0832, Sigma-Aldrich) was dissolved in saline to a concentration of 0.5 mg/ml. Silicon tubes were implanted for remote CNO injection. The tip of a 30 cm-long silicon tube was inserted 1 cm into the peritoneal cavity and sutured to the abdominal wall. The other end of the silicon tube was placed outside the body through an incision in the neck, and all incisions were sutured. All animals were then housed individually for a recovery period of at least 7 days. CNO was administered to each mouse (0.3 ml/30 g body weight) through the silicon tube. Injections were done at 21:00 and at 13:00.

### Sleep recordings

An electrode for EEG and EMG recording was implanted in the skull of each mouse as decscribed previously (Hara et al., 2001). The three arms of the electrode for EEG recording were placed ~2 mm anterior and 2 mm to the right, 2 mm posterior and 2 mm to the right, and 2 mm posterior and 2 mm to the left of the bregma. Stainless steel wires for EMG recording were sutured to the neck muscles of each mouse bilaterally, and each electrode was glued solidly to the skull. After the recovery period, animals were moved to a recording cage placed in an electrically shielded and sound attenuated room. A cable for signal output was connected to the implanted electrode and animals were allowed to move freely. Signals were amplified through an amplifier (AB-611J, Nihon Koden, Tokyo) and digitally recorded on a computer using EEG/EMG recording software (Vital recorder, Kissei Comtec). Animals were allowed at least 7 days to adapt to the recording conditions prior to any EEG/EMG recording session. Following the adaptation period, each animal was intraperitoneally administered both CNO and saline on separate experimental days with a 3-day interval. The order of injection was randomized. EEG/EMG data were evaluated and staged for 3 h after administration. Data acquired on the day of saline administration were used as control. We analyzed FFT spectra of NREM period in 1–2 h epoch of saline- or CNO-injected mice (*N* = 14). Power spectral analysis of EEG signals was performed using custom FFT software.

### Immunohistochemistry

To confirm GABAergic-specific expression of Cre recombinase activity in the POA of *Gad67-Cre* mice, we crossed them with Rosa26-tdTomato tracer mice (B6; 129S6-Gt(ROSA)26Sortm9(CAG-tdTomato)Hze/J, Jackson Laboratory #007905). Mice at 8 weeks of age were deeply anesthetized with sodium pentobarbital and then fixed by intracardiac perfusion with 4% paraformaldehyde. Then, the brain was post-fixed for 24 h in the same fixative and cryoprotected by immersion in 30% sucrose for 2 days. Cryostat sections (40-μm thick) of the brains were incubated for 1 h in 0.1 M phosphate buffer containing 1% bovine serum albumin and 0.25% Triton-X-100, and incubated overnight at 4°C with rabbit anti-GAD65/67 antibody (Uchigashima et al., 2007) in the same solution. After three washes in the same solution, the sections were incubated with goat anti-rabbit IgG conjugated with Alexa 488 (*Invitrogen*, Carlsbad, CA) for 90 min at room temperature. After three washes in 0.1 M phosphate buffer, the sections were mounted on glass slides and cover-slipped. Slides were examined with a laser-confocal microscope (Olympus FV10i, Olympus, Japan).

To detect monoaminergic and cholinergic neurons, we used mouse anti-tryptophan hydroxylase (TPH) antibody (Sigma, T0648, 1:200), guinea pig anti-histidine decarboxylase (HDC) antibody (PROGEN Biotechnik Gmbh, No.16046, 1:4,000), rabbit anti-tyrosine hydroxylase (TH) antibody (Millipore, AB152, 1:2,000), and goat anti-choline acetyltransferase (ChAT) antibody (Millipore, Ab144D, 1:100). As a second antibody, Alexa Fluor 594-goat anti-mouse IgG (Molecular Probes, 1:800), Alexa Fluor 594-goat anti-guinea pig IgG (Molecular Probes, 1:800) were used.

To detect Fos immunoreactivity in orexin-expressing neurons, coronal sections were incubated overnight with rabbit anti-cFos antibody Ab-5 (Calbiochem, 1:10000) and guenia pig anti-orexin antibody in 0.1 M phosphate buffer containing 1% bovine serum albumin and 0.25% Triton X-100. The primary antibody was localized with the avidin-biotin system (Vector). Bound peroxidase was visualized by incubating sections with 0.01 M imidazole acetate buffer containing 0.05% hydrogen peroxide and 2.5% nickel ammonium sulfate, resulting in a black reaction product in the nuclei. The sections were then incubated with anti-guenia pig IgG and then with the avidin-biotin-peroxidase complex as described above. Nickel sulfate was omitted from the final incubation, resulting in a golden brown reaction product in the cytoplasm. The numbers of cFos-positive and -negative orexin-containing neurons were counted in coronal sections throughout the hypothalamic region by a single examiner who was blinded to the treatment conditions, using a Keyence BZ-9000 microscope (Keyence, Japan). Cells were counted on both sides of the brain in consecutive 40-μm sections. Orexin neuron activity was scored as the percentage of double-labeled cells per animal.

### *In situ* hybridization

Double *in situ* hybridization was performed according to procedures previously described (Mieda et al., 2006). For double *in situ* hybridization, each combination of two antisense riboprobes labeled with either fluorescein-UTP (Gad1) or digoxygenin-UTP (GFP) was hybridized to sections simultaneously. Following the chromogenic reaction of the first color (blue) obtained with anti-digoxygenin-alkaline phosphatase (AP) Fab fragments, 5-bromo-4-chloro-3-indolyl phosphate (Roche) and nitroblue tetrazolium (Roche), sections were rinsed three times with TBS, treated twice with 0.1M glycine pH 2.2; 0.1% Tween 20 for 5 min, washed, and then incubated with anti-fluorescein-alkaline phosphatase (AP) Fab fragments. For the chromogenic reaction of the second color (orange), 5-bromo-4-chloro-3-indolyl phosphate (Roche) and 2-[4-iodophenyl]-3-[4-nitrophenyl]-5-phenyl-tetrazolium chloride (Roche) were used. Antisense riboprobes were synthesized from plasmids containing GFP and mouse Gad1 (NM_008077, nucleotides 281-821) cDNAs.

### Statistical analysis

Data were expressed as mean ± s.e.m. Two-way analysis of variance (ANOVA) followed by Bonfferoni correction as a *post-hoc* test or Student's *t*-test using IBM SPSS Statistics ver.19 was used for comparison among the various treatment groups. Differences were considered significant at *p* < 0.05.

## Results

### Pharmacogenetic selective stimulation of POA GABAergic neurons increased NREM sleep

**Figure 1:**
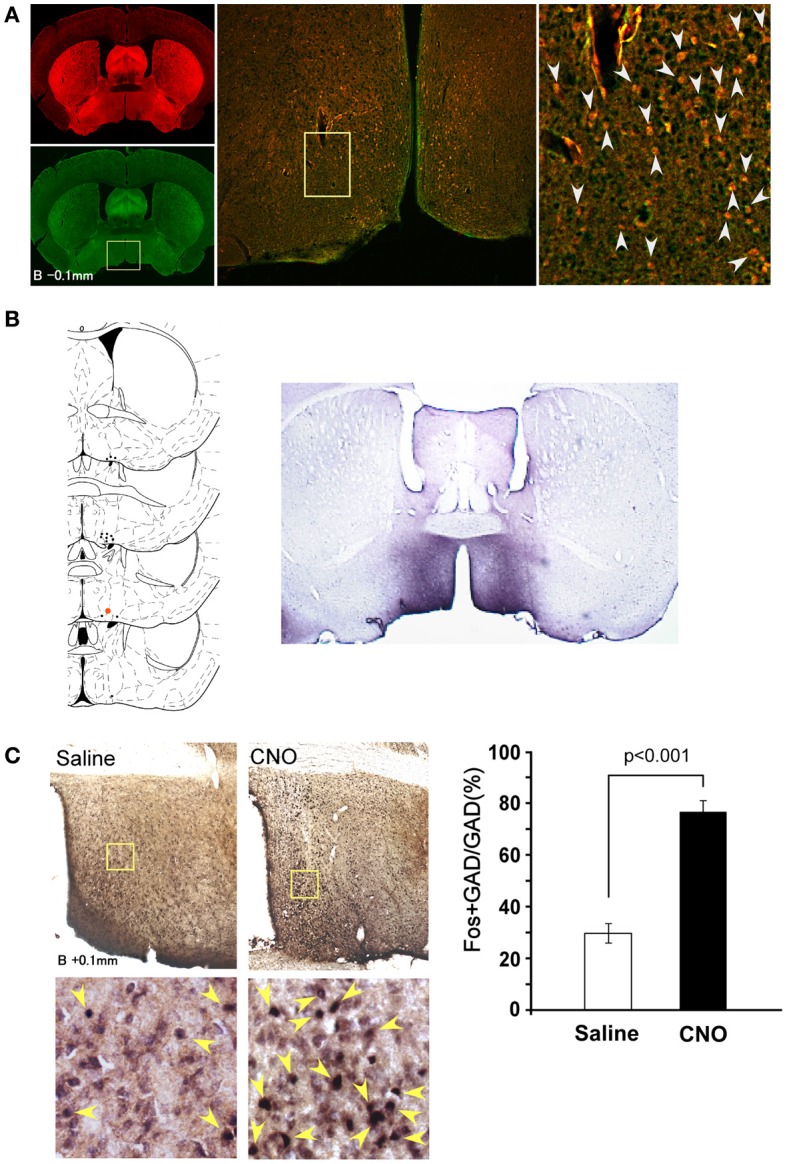
**Selective pharmacogenetic stimulation of POA neurons. (A)** Gad67-Cre mice were crossed with Rosa26-tdTomato reporter mice (see method) to confirm GABAergic neuron-specific expression of Cre recombinase. A representative image of the POA of the *Gad67-Cre; Rosa26-tdTomato* mice is shown (Bregma-0.1 mm). Left panels, upper, tdTomato fluorescence. Lower, same section stained with Gad65/67 anti-body. Middle panel, merged image of rectangular region in the left panel. Right panel: High power view of rectangular region in central panel. Arrowheads show the colocalization of tdTomato fluorescence and Gad65/67 immunoreactivity. **(B)** Left, Virus injection sites are shown by dots. Injection site for right panel image is shown by a red dot. Right, Representative image of HA-like immunoreactivity observed in POA region of Gad67-Cre mice injected with *AAV-DIO-HAhM3Dq* to express hM3Dq fused with HA tag. **(C)** Activation of POA neurons in Gad67-Cre mice expressing hM3Dq by CNO. Left panels, upper, representative images of double-immunostaining with anti-Fos and anti-Gad 65/67 in the POA region after administration of saline (left) or CNO (right) at 21:00. The brain was fixed at 23:00. Left panels, lower, high power view of the rectangular regions shown in upper panels. Arrowheads show the colocalization of Fos (nuclei) and Gad65/67 (cytoplasm). Right panel, Number of fos-immunoreactive GABAergic neurons in POA after treatment with saline or CNO (*N* = 4 and 4, respectively).

Before examining the connectivity between POA GABAergic neurons and orexin neurons, we confirmed whether specific stimulation of GABAergic neurons in the POA affects sleep/wakefulness states in mice. We applied the DREADD technology (Armbruster et al., 2007; Sasaki et al., 2011), to phamacogenetically manipulate the activity of POA GABAergic neurons. To express hM3Dq in GABAergic neurons in the POA, we injected *AAV-DIO-HAhM3Dq* into the POA of *Gad67-Cre* mice, in which GABAergic neurons specifically express Cre recombinase (Wu et al., 2011). GABAergic specific expression of Cre recombinase in the POA of *Gad67-Cre* mice was confirmed by crossing them with *ROSA26-tdTomato* mice (containing *tdTomato* gene preceded by a transcriptional blocker flanked with lox-P sites) (Figure 1A). We confirmed virtually all tdTomato-expressing neurons were positive for Gad65/67 immunoreactivity (95.4%). After injection of *AAV-DIO-HAhM3Dq*, we implanted thin silicone tubes into the peritoneal space of *Gad67-Cre* mice so that we could administer clozapine-N-oxide (CNO), the synthetic ligand for hM3Dq, with minimal disturbance. Fourteen days after virus injection, we administered CNO to mice.

Fourteen days after the virus injection, we administered CNO or saline intraperitoneally to *Gad67-Cre* mice expressing hM3Dq at 13:00 (light period) or 21:00 (dark period). The sleep/wakefulness states of these mice were monitored by simultaneous EEG/EMG recording. As a control, we treated the same mice with saline on separate experimental days. Each mouse was administered CNO or vehicle using a randomized crossover design at an interval of 3 days.

After the recording, mice were subjected to immunostaining with anti-HA antibody. We observed expression of HA-immunoreactivity in the POA region of most mice (Figure 1B). We injected the virus in 62 mice, and only used data obtained from 14 mice in which the existence of HA-positive cell bodies was limited within the POA. In many cases, we observed expression of HA-positive cells outside the POA, including the basal forebrain regions, such as the horizontal nucleus of the diagonal band. Therefore, we gathered data from 14 mice, in which HA-immunoreactivity was confined in the POA.

After the EEG/EMG recordings, we injected CNO (*n* = 8) or saline (*n* = 6) into *Gad67-Cre* mice expressing hM3Dq at 21:00, sacrificed and fixed them at 23:00. Hypothalamic slices of these mice were examined by double staining with anti-Fos and anti-Gad65/67 antibodies to assess the activity of POA neurons (Figure 1C).

We observed an approximately 2.5-fold increase in Fos-positive GABAergic neurons in the whole POA of the CNO-injected group as compared with the vehicle-injected group (29.7 ± 3.7% vs. 76.6 ± 4.3%, *p* < 0.001) (Figure 1C). These observations demonstrate that the DREADD system used in this study appropriately stimulates the activity of POA neurons.

**Figure 2:**
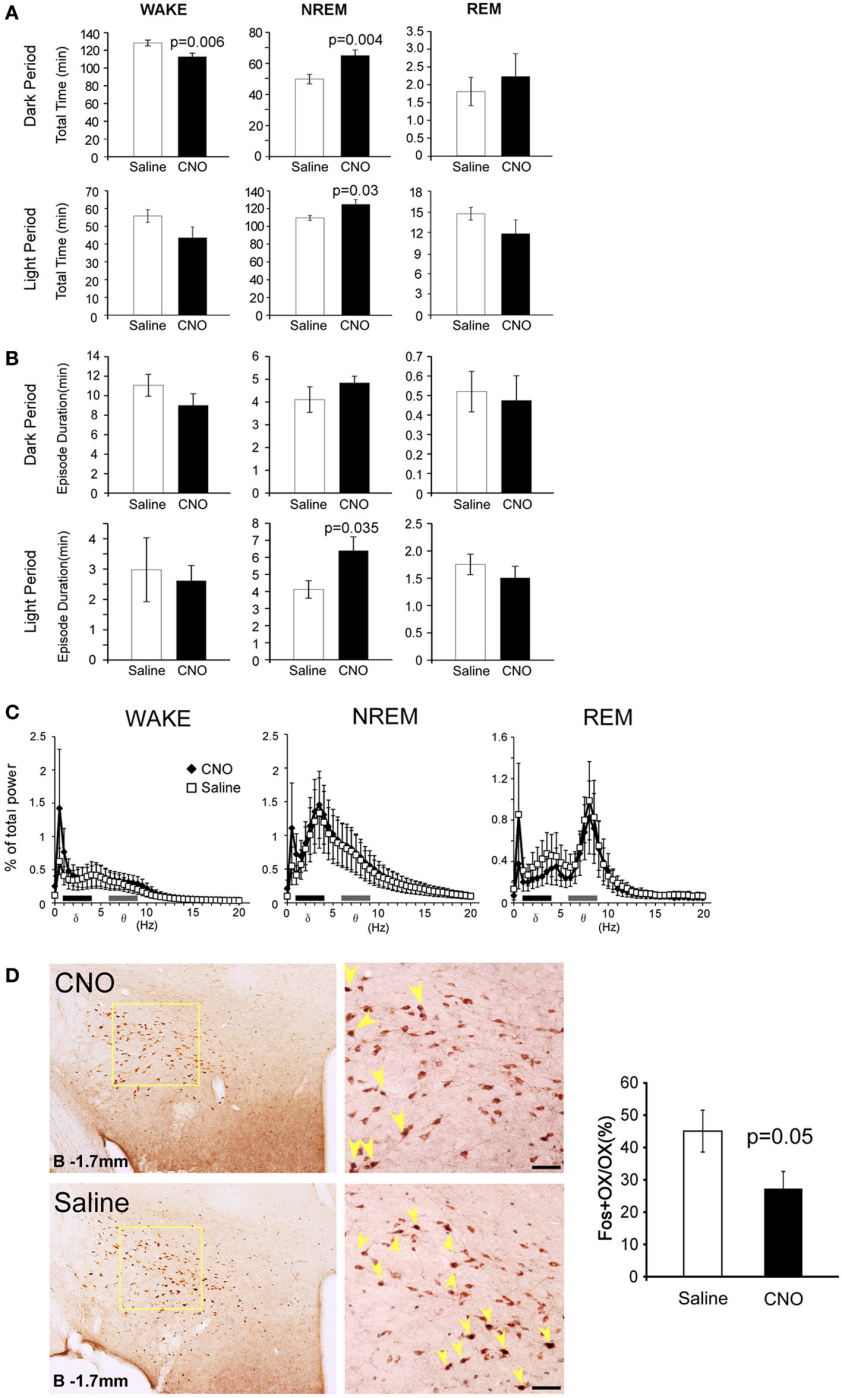
**Specific pharmacogenetic stimulation of GABAergic neurons in the POA increased NREM sleep amount. (A)** Total time of wakefulness (WAKE), NREM sleep and REM sleep for 3 h after CNO (or saline) administration at 21:00 (upper panels, Saline *n* = 14, CNO *n* = 13) and at 13:00 (lower panels, Saline *n* = 7, CNO *n* = 7). **(B)** Episode duration of WAKE, NREM sleep, and REM sleep for 3 h after CNO (or saline) administration at 21:00 (upper panels, Saline *n* = 14, CNO *n* = 13) and at 13:00 (lower panels, Saline *n* = 7, CNO *n* = 7). **(C)** EEG power density of WAKE, NREM sleep and REM sleep in 1–2 h time window after the administration of CNO or saline at 21:00 (Saline *n* = 14, CNO *n* = 13). EEG power density is shown as the mean percentage of total EEG power ± s.e.m. **(D)** Representative images of fos expression in orexin neurons, as shown by double staining of the LHA of Gad67-Cre mice 2 h after injection of saline (*n* = 5) or CNO (*n* = 8) at 21:00. Scale bars, 50μm. Right panel, ratio of Fos-positive orexin neurons after injections of saline or CNO. Arrowheads show colocalization of Fos (nuclei) and orexin (cytoplasm).

EEG/EMG analyses found that the percent of wakefulness during 3 h after CNO administration was significantly shorter (128.3 ± 3.3min vs. 112.7 ± 4.1 min, *p* = 0.006), while NREM time was longer in CNO-treated conditions than in saline-injected control conditions in the dark period (49.9 ± 3.0 min vs. 65.1 ± 3.1 min, *p* = 0.004) (Figure 2A). A significant increase of NREM sleep was also observed for 3 h after administration in the light period (13:00). We observed an increase in NREM episode duration when CNO was administered in the light period (Figure 2B). Although we also observed similar tendency when CNO was administered in the dark period, the difference was not statistically significant. The power density of EEG of each episode in the CNO-administered group in the dark period showed no difference from that in the vehicle-administered group (Figure 2C).

These results suggest that, consistent with previous non-specific electrical and chemical stimulation studies of the POA, specific stimulation of POA GABAergic neurons results in a decrease of wakefulness time, accompanied by increased NREM sleep time. We did not observe a significant difference in REM sleep time between the CNO-injected and control groups (Figure 2A).

We next examined the effect of stimulation of POA GABAergic neurons on orexin neuronal activity by Fos-immunostaining. After CNO or saline was injected at 21:00, the brains were fixed at 23:00, and subjected to double staining with anti-orexin and anti-Fos antibody. We observed decrease in number of double positive cells (45.1 ± 6.5% vs. 27.3 ± 5.3%) (Figure 2D), although it was unknown whether the inhibition was directly mediated by POA GABAergic neurons or rather resulted from increased amount of sleep, because orexin neuronal activity was shown to correlate with the amount of wakefulness (Estabrooke et al., 2001).

### POA GABAergic neurons send innervations to regions implicated in the regulation of sleep/wakefulness states

**Figure 3:**
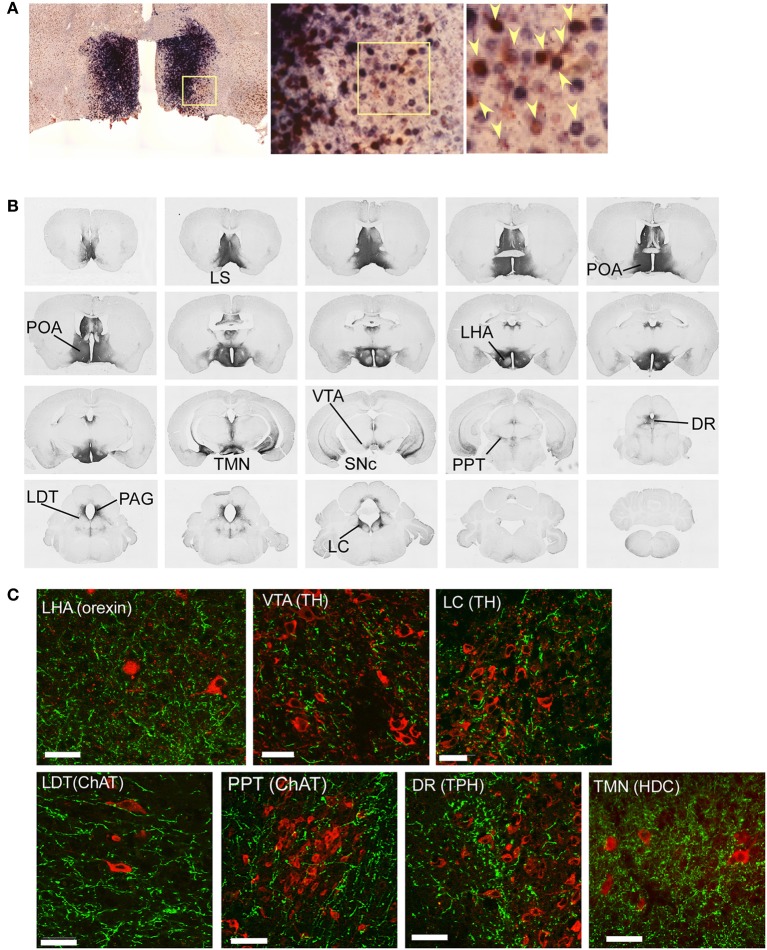
**Mapping of projection sites of the POA GABAergic neurons. (A)** Expression of *ChR2-eYFP* mRNA (blue) and *Gad67* mRNA (red) in the POA of *Gad67-Cre* mice after bilateral injection of *AAV-DIO-hChR2(H134R)-eYFP* into the POA. Almost all (>95%, *n* = 4) *ChR2-eYFP* mRNA-positive neurons also expressed *Gad67* mRNA. Arrowheads show co-localization of *ChR2-eYFP* mRNA and *Gad67* mRNA. **(B)** After injection of *AAV-DIO-hChR2(H134R)-eYFP* into the POA of *Gad67-Cre* mice, the brain was subjected to histological analysis. Representative images show localization of ChR2-positive fibers from rostral to caudal in Gad67-Cre mouse brain coronal sections revealed by immunohistochemical staining using anti-GFP antiserum. **(C)** Images of single confocal planes showing ChR2-positive axonal fibers observed in the LHA, VTA, LC, LDT, PPT, DR, and TMN. Slices were stained with anti-GFP (green) and designated antibodies (red), including anti-orexin, anti-TH, anti-ChAT, anti-TPH and anti-HDC antibodies. Scale bars: 40 μm.

We next examined whether POA GABAergic neurons directly innervate orexin neurons. We injected *AAV-DIO-hChR2(H134R)-EYFP* into the POA of *Gad67-Cre* mice, because ChR2 works well as an anterograde tracer (Harris et al., 2012). Because ChR2-eYFP is distributed in axons and dendrites, it is difficult to observe cell bodies of neurons that express ChR2-eYFP, we examined the expression pattern of *ChR2-eYFP* mRNA in the POA by double label *in situ* hybridization to detect the original cell bodies that expressed *ChR2-eYFP* mRNA and *Gad67* mRNA (Figure 3A). *ChR2-eYFP* mRNA-expressing cells were widely spread within the POA. Almost all these *ChR2-eYFP* mRNA-positive neurons also expressed *Gad67* mRNA. We injected the virus into 27 mice, and selected four mice in which *Gad67* mRNA expression was confirmed to be restricted within the POA.

Following the specific expression of ChR2 in GABAergic neurons in the POA of *Gad67-Cre* mice, we observed a network of ChR2-containing axons in the brain by immuostaining the YFP-positive fibers (Figure 3B, Table 1). We found abundant ChR2-eYFP-positive fibers in many of known arousal-related regions in the hypothalamus and brain stem, including the LHA, ventral tegmental area (VTA), substantia nigra pars compacta (SNc), TMN, LC, and LDT (Figure 3B). The projecting fibers seemed to avoid the main part of the dorsal raphe (DR), and density of the fibers in the PPT was sparser than other arousal-related regions, although we found considerable numbers of YFP-positive fibers in these regions (Figure 3C). Double staining studies suggested that these fibers made apposition to LC noradrenergic neurons, PPT cholinergic neurons, DR serotonergic neurons and TMN histaminergic neurons (Figure 3C). When we infected *AAV-DIO-hChR2(H134R)-EYFP* into the POA unilaterally, most of (>95%) the axonal projections were found in the ipsilateral side (not shown).

**Table 1:** **Projection sites of POA GABAergic neurons**.

**Cell group**	**Relative density**
**I. FOREBRAIN**	
** A. Isocortex**	
I	±
II	±
III	±
IV	±
V	±
VI	±
Claustrum	++
Endoperiform nucleus	++
** B. Hippocampal formation**	
1. Entorhinal area	+
2. Subculum	+
3. CA1	+
4. CA2	+
5. CA3	+
6. Dentate	±
7. Induseum griseum	−
** C. Amygdala**	
1. Medial nucleus	++
2. Amygdalohippocampal area	−
3. N. lat. Olfactory tract	−
4. Anterior amygdaloid area	−
5. Central nucleus	−
6. Lateral nucleus	−
7. Basolateral nucleus	+
8. Basomedial nucleus	+
9. Intercalated nuclei	+
10. Cortical nucleus	+
** D. Septum**	
1. Lateral nucleus	+++
Dorsal part	+++
Intermediate part	+++
Ventral part	+++
2. Medial nucleus	+++
3. Bed n. stria terminalis	+++
Rosteromedial resion	+++
Rosterolateral resion	+++
Posterodorsal resion	+++
Posteroventral resion	+++
4.Septofimbrial nucleus	+++
5. Subfornical organ	+++
6. Bed n. anterior commissure	+++
** E. Basal ganglia**	
1. Caudoto putamen	−
2. Glabus pallidus	−
3. Substantia nigra, Conpact part	+++
4.Substancia nigra, reticular part	−
5. Subthalamic mucleus	++
** F. Thalamus**	
1. Medial habenula	+++
2. Lateral habenula	++
3. Anterior group	
Anteroentral n.	+++
Anteromedial n.	++
Anterodorsal n.	++
Interanterolmedial n.	−
Interamediodorsal n.	+
4. Mediodorsal nucleus	
Medial part	+++
Central part	+++
Lateral part	−
5. Lateral groupe	
Lateral dorsal n.	−
Lateral posterior n.	−
6. Midline group	
Paraventricular n.	+++
Paratenial n.	+
Central medial n.	+++
Centrolateral n.	−
Rhomboid n.	−
N. reuniens	−
7. Posterior complex	+
8. Medial geniculate n.	−
9. Lateral geniculate n.	−
10. Intralaminar nuclei	−
11. Reticular nucleus	−
12. Zona incerta	+
13. N.firlds of Forei	±
** G. Hypothalamus**	
1. Periventricular zone	+++
Median preoptic n.	+++
Anteroventral periventricular n.	+++
Preoptic periventricular nucleus	+++
Suprachiasmatic n.	+++
Supraoptic nucleus	+++
Paraventricular n.	+++
Parvicellular part, post	+++
Magnocellular part	+++
Periventricular nucleus	+++
Arcuate nucleus	+++
Posterior periventricular n.	++
2. Medial zone	
Medial preoptic area	+++
Medial preoptic n.	+++
Anterior hypothalamic n.	+++
Retrochiasmatic area	+++
Ventromedial n.	+++
Dorsomedial n.	+++
Tuberomammillary n.	+++
Supramammillary n.	+++
Lateral mammillary n.	+++
Medial mammillary n.	+++
3. Lateral zone	
Lateral preoptic area	+++
Lateral hypothalamic area	+++
Posterior hypothalamic area	+++
**II. BRAIN STEM**	
** A. Sensory**	
1. Visual	
Superior colliculus	−
Parabigeminal n.	−
Pretectal resion	
Olivary n.	−
N. optic tract	−
Anterior n.	−
Posterior n.	−
Medial pretectal area	−
N. posterior commissure	−
2. Somatosensory	
Mesencephalic n. (5)	+
Principal sensory, n. (5)	−
Spinal n.	−
Gracile n., dorsal	−
3. Auditory	
Cochlear nuclei	
Dorsal	−
Ventral	−
N. trapezoid body	±
Superior olive	+
N. lateral lemniscus	−
Inferior colliculus	−
Exterminal	−
Dorsal	−
Central	−
N. brachium inf. Coll.	−
4. Vestibular	−
5. Visceral	
N. solitary tract	−
Area postrema	−
Parabranchial n.	
Lateral	+
Medial	+
** B. Motor**	
1. Eye	
Oculomotor(3)	+
Edinger-westphal nucleus	++
Trochlear(4)	+
Abducens(6)	−
2. Jaw	
Motor n. (5)	−
3. Face	
Facial n. (7)	±
4. Pharynx/larynx	
N. ambiguus	−
5. Tongue	
Hypoglossal. n.(12)	−
6. Viscera	
Dorsal motor n. (10)	+
** C. Reticular core (including central gray and raphe)**	
1. Periaqueductal gray-assoc. w/PAG	
Interstitial n. of cajal	−
Dorsal tegmental n.	−
Laterodorsal teg. N.	+++
Barrington's n.	+++
Locus coeruleus	+++
2. Raphe	
Interfascicular n.	+++
Rostral linear n.	+++
Dorsal raphe	+++
Median raphe	+++
N. raphe pontis	+++
N. raphe magnus	+++
N. raphe pallidus	+++
3. Interpeduncular n.	
Rostral subnucleus	±
Apical subnucleus	±
Dorsomedial subnucleus	−
Lateral subnucleus	±
Intermediate subnucleus	+
Central subnucleus	+
4. Reticular formation	
Central teg. field	+
Peripeduncular n.	−
Pedunculopontine n.	++
Cuneiform n.	++
Pontine reticular	+
Parvocellular ret. Firld	+
Gigantocellular ret.	+
Lat. paragigantocellular	+
Intermediate ret. field	−
Paramedian reticular n.	−
** D. Pre- and postzerebellar**	
1. Pontine gray	−
2. Tegmental reticular n.	−
3. Lateral reticular n.	−
4. Red nucleus	−
5. N.Roller	−
6. Prepositus hypoglossal nucleus	−
**III. CEREBELLUM**	
1. Flocculus	−
2. Other parts	−

We attempt to grade the density of fiber-like structures in the sections into five categories according to Nambu et al. (1999); dense (+++); moderately dense (++); sparse (+); very sparse (−).

We found prominent projections to the LHA (Figures 3B,C), in which orexin neurons are localized. Double staining of LHA slices with anti-GFP and anti-orexin antibodies showed that most orexin neurons in the LHA were densely surrounded by rich ChR2-eYFP fibers (Figure 3C). This suggests that POA GABAergic neurons send innervations to orexin neurons in the LHA.

### Detecting spike-mediated GABA release from axons of POA neurons onto orexin neurons

**Figure 4:**
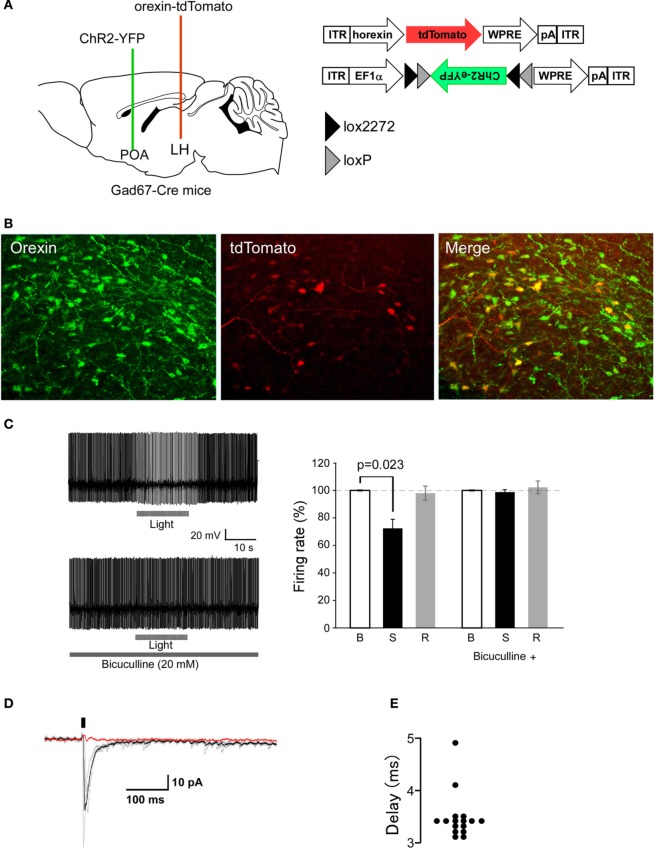
**Optogenetic stimulation of POA GABAergic axons rapidly decreases orexin neuron firing. (A)** Strategy of this study. We simultaneously injected *AAV-horexin-tdTomato* into the LHA and *AAV-DIO-hChR2(H134R)-eYFP* into the POA. **(B)** Identification of orexin neurons in the LHA by expression of tdTomato in these neurons after injection of *AAV-horexin-tdTomato*. Left: Coronal section at bregma −2.1 mm showing distribution of orexin neurons stained by anti-orexin antiserum (green). Center: Cells with red fluorescence of tdTomato. Right: Merged image **(C)** Typical electrical current-clamp recording of orexin neuron. Left: Example of changes in firing of orexin neurons induced by optical stimulation in the absence (top trace) and presence (bottom trace) of bicuculline (20 μM). We observed similar responses in three out of eight cells. Right: Group data for response and recovery at 10 Hz stimulation, expressed at % change in firing before **(B)**, during (S) and after (R) light stimulation. **(D)** Optogenetically induced IPSCs in orexin neurons with and without bicuculline (20 μM). Holding potential was −60 mV. Black bar indicates light stimulus (5 ms, 1 Hz). Individual responses without bicuculline are shown in gray, average without bicuculline is in black, and average with bicuculline is in red. **(E)** Times after light-on to IPSPs onset are plotted for individual stimulations.

To test if stimulation of these axons modulates activity of orexin neurons, we performed whole-cell patch-clamp recordings from orexin neurons during optical stimulation of ChR2-eYFP-containing axons. To make identification of orexin neurons easy, we expressed tdTomato specifically in orexin neurons by AAV-mediated gene transfer (*AAV-horexin-tdTomato*). We used the human *prepro-orexin* promoter (Sakurai et al., 1999) to express tdTomato specifically in orexin neurons (Figure 4A). Immunolabeling confirmed that virtually all (>97%, *n* = 3) tdTomato-expressing neurons also contained detectable orexin-like immunoreactivity, suggesting highly specific expression of tdTomato in orexin neurons after injection of AAV (Figure 4B), although there were many orexin neurons that were negative for tdTomato fluorescence, suggesting incomplete penetrance of the virus-mediated expression.

To examine the connectivity between POA GABAergic neurons and orexin neurons, we simultaneously injected *AAV-DIO-hChR2(H134R)-EYFP* into the POA and *AAV-horexin-tdTomato* in the LHA of *Gad67-Cre* mice. 14 days after the injection, we prepared acute LHA slices and made patch clamp recordings from red fluoresced cell. We then stimulated axons with light emitting diode (LED) light of 470 nm in a 90-μm diameter window surrounding recorded orexin neurons. When orexin neurons were recorded under current-clamp with zero holding current, the light flashes slowed firing rate (Figure 4C). After recording for several minutes without stimuli, we applied 20-ms light stimuli (10 Hz). Since a previous study suggested the firing rates of sleep-active neurons in the POA ranged between about 5 and 20 Hz (Takahashi et al., 2009), we tried stimulation frequencies of 6, 10, and 20 Hz, and found that frequencies above 20 Hz evoked strong inhibition, but this lasted a very short time (for only about 2 s). Because 10 Hz stimulation caused strong and long-lasting inhibition of orexin neurons (data not shown), we used 10 Hz stimulation throughout this study. The inhibition of orexin neuron firing was completely abolished by a specific GABA_*A*_ antagonist, bicuculline (Figure 4C).

Optical stimulation of ChR2-axons located near orexin neurons produced fast inhibitory post-synaptic currents (IPSCs) in these cells (Figure 4D). Orexin neurons from slices prepared from mice without ChR2 expression did not show any membrane responses to the same light flashes (*n* = 10, data not shown), confirming that without ChR2, our optical stimulation does not affect synaptic input to orexin neurons. In ChR2-expressing slices, the delay between flash onset and post-synaptic response was 3.4 ± 1.3 ms (Figure 4E). This short delay suggests that it is likely that GABAeric fibers extending from the POA directly inhibit orexin neurons. These observations show that GABAergic axons originating from POA neurons modulate orexin neurons via GABA_A_ receptor-mediated synaptic transmission.

## Discussion

Extracellular recording studies have demonstrated cells in the POA that display elevated firing rates during sleep with attenuated firing during wakefulness (Findlay and Hayward, 1969; Kaitin, 1984). The discharge rate of these “sleep-active” neurons increased several seconds prior to NREM sleep onset as defined by EEG changes. These observations suggest that the POA plays an important role in the initiation and maintenance of sleep. Approximately 80% of sleep-active neurons in the VLPO also contain the neuropeptide galanin, which is highly colocalized with GABA in VLPO neurons (Sherin et al., 1998; Gaus et al., 2002). The number of Fos and GAD-double positive neurons in both the MnPN and the VLPO was shown to be positively correlated with the amount of preceding sleep (Gong et al., 2004).

These POA sleep-regulatory neurons were shown to be activated by adenosine through both direct and indirect actions. Adenosine caused A_1_ receptor-mediated suppression of spontaneous IPSPs in rat VLPO neurons recorded *in vitro* (Chamberlin et al., 2003). Moreover, an adenosine A_2A_ receptor agonist evoked direct excitatory effects on a subset of rat VLPO neurons (Gallopin et al., 2005). Furthermore, perfusion of an A_2A_ agonist into the POA in rats promoted sleep (Satoh et al., 1999). These mechanisms have been thought to play an important role in homeostatic regulation of sleep through actions of adenosine.

A recent extracellular recording study suggested widespread distribution of sleep-active neurons within the whole POA (Takahashi et al., 2009), so it is necessary to genetically target the cell types being manipulated. In this study, we expressed hM3Dq or ChR2 broadly in GABAergic neurons in the POA. Firstly, we confirmed that specific stimulation of POA GABAergic neurons leads to an increase of NREM time (Figure 2). Sasaki et al. (2011) reported the same or even more NREM induction by hM4D-mediated inhibition of orexin neurons. This was unexpected, because the activation of POA GABA neurons should suppress more wake-active neurons widely throughout the brain. One possible reason why the effects were not so strong in this study is that we activated large numbers of GABAergic neurons in the POA. It is known that only limited number of GABAergic neurons in the POA would become active during sleep. However, in this study, larger numbers of the GABAergic neuorns in the POA, including GABAergic interneurons, might be activated. Some population of GABAerigic neurons might rather inhibit sleep-active neurons to counteract direct activation of these cells by CNO.

We also expressed ChR2-eYFP selectively in GABAergic neurons in the POA of *Gad67-Cre* mice. This allowed us to trace axonal fibers of these cells, and perform fast electrical control of action potential firing of these fibers with light (Petreanu et al., 2007). Firstly, we examined the pattern of axonal projections by staining eYFP with an anti-GFP antibody. This revealed that GABAergic neurons in the POA send projections to arousal-regulating regions in the brain stem, including the LC, DR, LDT/PPT, and TMN (Figures 3B,C, Table 1). Double immunofluorescence study further suggested that these axonal fibers make appositions to orexin neurons in the LHA as well as other arousal-related neurons including TH-positive, noradrenergic cells in the LC, serotonergic cells in the raphe nuclei, cholinergic cells in the LDT, and histaminergic cells in the TMN (Figure 3C).

We next examined the effect of optogenetic stimulation of ChR2-positive fibers around orexin neurons in the LHA (Figure 4). In the stimulation paradigms used here, blockade of GABA_*A*_ receptors completely abolished the post-synaptic effect of GABAergic axon stimulation, suggesting that release of other transmitters, such as galanin, was not sufficient to alter orexin neuron firing.

Previous studies as well as our present findings suggest that POA GABAergic neurons send rich innervations to multiple brain regions, including monoaminergic/cholinergic nuclei in the brain stem, which fire at a rapid rate during wakefulness, slow down during NREM sleep, and cease firing during REM sleep, and are implicated in maintenance of wakefulness (Figure 3B). This means it is difficult to speculate on the relative contribution of the inhibitory action on orexin neuronal activity in increasing NREM sleep time. However, because specific pharmacogenetic or optogenetic inhibition of orexin neurons was shown to increase NREM sleep (Sasaki et al., 2011; Tsunematsu et al., 2011), it is possible to speculate that POA GABAergic neuron-mediated NREM sleep promotion might be at least partly through the inhibition of orexin neurons. Further studies, including optogenetic/pharmacogenetic activation/inhibition of POA GABAergic neurons *in vivo* in orexin-deficient animals, will be required to address this.

### Conflict of interest statement

The authors declare that the research was conducted in the absence of any commercial or financial relationships that could be construed as a potential conflict of interest.
